# VirGrapher: a graph-based viral identifier for long sequences from metagenomes

**DOI:** 10.1093/bib/bbae036

**Published:** 2024-02-11

**Authors:** Yan Miao, Zhenyuan Sun, Chenjing Ma, Chen Lin, Guohua Wang, Chunxue Yang

**Affiliations:** College of Computer and Control Engineering, Northeast Forestry University, Hexing Road, 150040, Heilongjiang Province, China; College of Computer and Control Engineering, Northeast Forestry University, Hexing Road, 150040, Heilongjiang Province, China; College of Computer and Control Engineering, Northeast Forestry University, Hexing Road, 150040, Heilongjiang Province, China; National Institute for Data Science in Health and Medicine, Xiamen University, Xiangannan Road, 361104, Fujian Province, China; College of Computer and Control Engineering, Northeast Forestry University, Hexing Road, 150040, Heilongjiang Province, China; College of Landscape Architecture, Northeast Forestry University, Hexing Road, 150040, Heilongjiang Province, China

**Keywords:** metagenome, long viral identification, graph neural network

## Abstract

Viruses are the most abundant biological entities on earth and are important components of microbial communities. A metagenome contains all microorganisms from an environmental sample. Correctly identifying viruses from these mixed sequences is critical in viral analyses. It is common to identify long viral sequences, which has already been passed thought pipelines of assembly and binning. Existing deep learning-based methods divide these long sequences into short subsequences and identify them separately. This makes the relationships between them be omitted, leading to poor performance on identifying long viral sequences. In this paper, VirGrapher is proposed to improve the identification performance of long viral sequences by constructing relationships among short subsequences from long ones. VirGrapher see a long sequence as a graph and uses a Graph Convolutional Network (GCN) model to learn multilayer connections between nodes from sequences after a GCN-based node embedding model. VirGrapher achieves a better AUC value and accuracy on validation set, which is better than three benchmark methods.

## INTRODUCTION

Viruses are the richest biological entities on the earth [1] and are widely existed in soil, marine, human body, etc. [2]. They replicate in host cells and play a very important role in controlling bacterial population size and altering host metabolism through interactions with host [3–5]. Especially in plants, virus infection may result in changes in plant physiological functions, including delayed growth and development, inferior quality and decreased yield, leading to serious economic losses [6]. Next generation sequencing technology can generate a lot of sequences from a variety of environmental samples in a short period, constructing a metagenome [6, 7]. To analyze viral–host interactions [8] from metagenomic data and further analyze human diseases [9–12], such as colorectal cancer (CRC) [13–15] and inflammatory bowel disease [16], identifying viral sequences directly from metagenome is the very first step [17]. Because of the vast number of sequences and the low content of virus sequences in metagenomes [18], identifying viral sequences accurately becomes a challenge.

In the past few years, several deep learning-based methods have achieved significant improvements in accuracy in short viral sequence identification, such as RNN-VirSeeker [19], DeepVieFinder [20], PPR-Meta [21], Virtifier [22] and CHEER [23]. When they identify long viral sequences (>1000 bp), a long sequence has to be divided into a lot of non-overlapped short sequences, and then, these subsequences are input into their neural networks, respectively, to get their own viral scores. The average of these scores will be the final scores and is contributed to identify whether the long sequence is viral or not. Take Virtifier for an example, when identifying a query sequence longer than 500 bp, it will divide the long sequence into several non-overlapped subsequences of 500 bp. The last bases of the sequence shorter than 500 bp will be zero-padded to a single subsequence. Then, all subsequences are input to the 500-bp trained Virtifier one by one to get their own scores. The average of them is considered as the final score to determine if the query long sequence is virus.

However, when a long sequence is cut off, some useful regions may be separated at the same time. If those regions contribute more to the features of the long sequence, the operation of cutting off leads to losing significant information for identifying viruses [22]. Furthermore, there may be relationships between regions in different parts of a long sequence. When the short sequences generated from a long sequence were input into the deep learning model separately, those relationships were naturally omitted. The above factors have led to poor performance of deep learning methods in identifying long viral sequences. This may be one of reasons that Virtifier has a bad performance on identifying long viral sequences.

**Figure 1:**
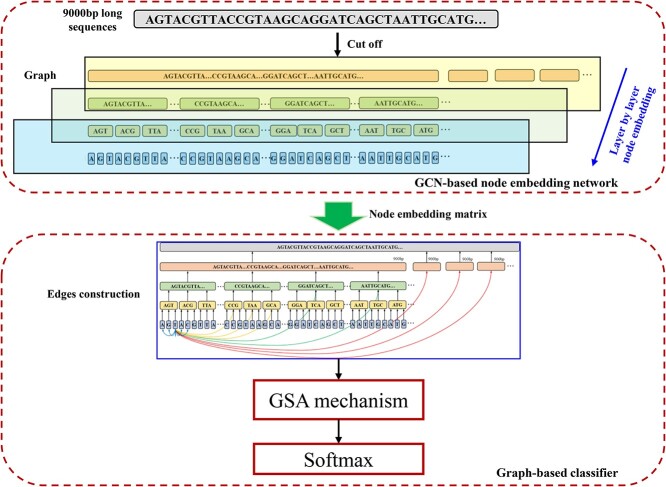
The workflow of VirGrapher.

In this paper, VirGrapher is proposed to improve the identification performance of long viral sequences from metagenomes by constructing relationships among short subsequences from long ones. VirGrapher is built based on a Graph Convolutional Network (GCN) [24] to learning multilayer connections between nodes from sequences after a GCN-based node embedding model. The workflow of VirGrapher is shown in Figure 1. A long sequence is firstly divided into several subsequences layer by layer. These subsequences are considered as nodes in a graph. A GCN-based model is used to learn these node embeddings layer by layer. Then, some multilayer connections are constructed to the graph, which is trained by the Graph Self-Attention (GSA) mechanism [1]. After being fully trained, VirGrapher achieves the AUC of 0.9604 and the accuracy of 0.9413 on validation set.

## MATERIALS AND METHODS

### Virus and host RefSeq genome datasets for training, validating and testing

About 13 274 virus RefSeq genomes (up to 1st October 2020) were downloaded from NCBI Virus (https://www.ncbi.nlm.nih.gov/l abs/virus/vssi/#/virus?SeqType_s=Genome&SourceDB_s=Re fSeq). Being combined with 4410 host RefSeq genomes from VirFinder [25], all of the genomes were jointly used to train VirGrapher. To train the vector representations of sequences from each level, all of sequences from the virus and host RefSeq genomes were split into a set of non-overlapped fragments with a length of 9000 bp, resulting 43 243 viral sequences and 51 991 host sequences, where sequences shorter than 9000 bp were zero-padded to 9000 bp. All 43 243 viral sequences and 43 243 host sequences subsampled randomly were established as embedding training dataset. Then, every 9000-bp sequences were split into 10 non-overlapped fragments with a length of 900 bp, namely embedding training dataset 1 (ETD_1). These 900-bp sequences were split into 100 90-bp subsequences without overlap, namely embedding training dataset 2 (ETD_2). The sequences from ETD_2 were split into 1000 non-overlapped 9-mer fragments, namely embedding training dataset 3 (ETD_3). Lastly, every 9-mer fragments in ETD_3 was split into a sequence of 9 bases, namely embedding training dataset 4 (ETD_4).

To obtain more sequences for training graph neural network, 300 000 viral sequences and 3 000 000 host sequences with a length of 9000 bp were randomly subsampled from the sequences longer than 9000 bp in virus and host RefSeq genomes. These 600 000 sequences were used as a training set to train the graph neural network in VirGrapher, and 10 000 viral sequences and 10 000 host sequences with a length of 9000 bp were also randomly subsampled to build a validation set to test the performance of trained VirGrapher.

To test the performance of VirGrapher on identifying long viral sequences from metagenomes, three real metagenomes were chosen as testing datasets: 1st CAMI Challenge Dataset 3 CAMI_high Dataset, 2nd CAMI Challenge Marine Dataset and a real human gut metagenome.

The first metagenome was downloaded from the 1st CAMI Challenge Dataset 3 CAMI_high Dataset (https://data.cami-challenge.org/participate). Sequences from the gold standard assembly of all five samples were first compared with NT (Nucleotide Sequence Database) from NCBI website using BLAST (Basic Local Alignment Search Tool) [26] with default parameters and these longer than 2000 bp were extracted from the comparison result. Then, these long sequences were firstly mapped to virus RefSeqs and the rest unmapped ones were then mapped to host RefSeqs by BLAST [26]. Sequences whose E-values lower than $10^{-5}$ were considered as viral sequences and host sequences, respectively. The blast comparison resulted 732 viral sequences and 2198 host sequences. The 2nd CAMI Challenge Marine Dataset (https://data.cami-challenge.org/participate) is a short and long read shotgun metagenome data from samples at different seafloor locations of a marine environment. All sequences from CAMI2 short read pooled gold standard assembly were preprocessed as what had been down for CAMI_high Dataset. After the blast comparison [12], 876 viral sequences and 15 941 host sequences longer than 2000 bp were collected as a metagenome dataset to test VirGrapher. The last metagenome is from a real human gut metagenomic sample, where reads are downloaded from the NCBI short-read archive (accession ID: SRA052203 [27]). The sequences in the sample were also processed according to the CAMI Challenge Marine Dataset. Using BLAST comparisons, 3769 viral sequences and 14 264 host sequences longer than 2000 bp were filtered as the real human gut metagenome.

### Construction of VirGrapher

#### GCN-based node embedding network

A two-layer GCN network is built to train word embedding vectors for long sequences according to the GCN-based sequence embedding model in DETIRE [28]. Four two-layer GCNs are trained separately by the four embedding training datasets ETD_1-4. When training GCN_1 using ETD_1, the parent sequences are 9000-bp sequences, and the subsequences are 900-mer sequences. The nodes between parent sequences and subsequences are initialized by one-hot vectors. In GCN_2, the parent sequences are 900-mer sequences and the subsequences are 90-mer sequences. The nodes of the parent sequences are initialized by the subsequence embedding vectors obtained from GCN_1, and the nodes of the subsequence are initialized by one-hot vectors. When training GCN_3 by ETD_3, the nodes of the 90-mer sequences are initialized by the subsequence embedding vectors obtained from GCN_2, and the nodes of the 9-mer subsequences are initialized by one-hot vectors. For GCN_4, the nodes of 9-mer parent sequences are initialized by the subsequence embedding vectors obtained from GCN_3, and the nodes of the subsequences are initialized by one-hot vectors. GCN_4 is trained by ETD_4. In this case, the embedding vector dimensions of the 900-bp sequences, the 90-mer sequences, the 9-mer sequences and the bases are all set to 30.

Finally, the graph is trained by an another two-layer GCN model before a softmax classifier. A cross-entropy loss [29] is used to calculate the errors between predictions and labels. The batch-size is set to 200. During training, we tried several different hyper-parameters. The loss dropped rapidly before 100 epochs and was stabilized at 200 epochs. After 200 epochs, the loss almost did not change. After 200 epochs of backpropagation by the Adam [30] optimization algorithm with a learning rate of 0.003, the vectors of each node in the second layer are the embedding vector for each parent sequence or subsequence.

#### Graph-based classifier

**Figure 2:**
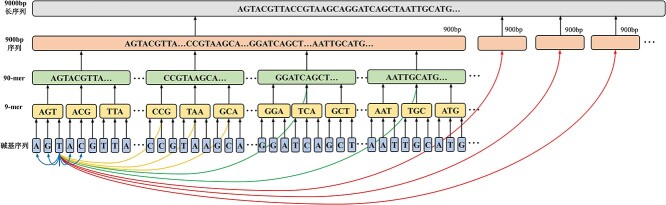
The graph established from a 9000-bp sequence.

Because of the insufficient linkage between short sequences existing widely in deep learning-based methods for identifying long viral sequences, a graph containing multilayer connections is constructed (Figure 2).

Sequences shorter than 9000 bp are zero-padded to 9000 bp. For sequences longer than 9000 bp, 9000 bp sequences are randomly extracted for identification. A 9000 bp sequence is divided into 10 900-bp sequences without overlapping. These 900 bp sequences are then divided into 10 non-overlapped 90-mer sequences. Each 90-mer sequence is further cut off into 10 9-mer sequences. Finally, every 9-mer sequence is separated into 9 bases. These subsequences are all considered as nodes in a graph. If a subsequence comes from a parent sequence, a ’direct edge’ is built between them (represented by a black line in Figure 2). To construct relationships between subsequences and reduce the distance of information transmission between them, ’local edges’ and ’segment edges’ are built. Direct edges are built between child and parent nodes. Local edges (called $E_{1}$ ) are created between each base and its 2k neighbors ($k$ is a hyperparameter which is set to 4). Segment edges connect base nodes and other nodes at different levels. The edges between each base node and 10 9-mer nodes to their right are represented by yellow lines (called $E_{2}$ ). The edges between each base node and six 90-mer nodes to their right excluding $E_{2}$ edges are represented by green lines (called $E_{3}$ ). The red lines represent the edges between each base node and three 900 bp nodes to their right except $E_{2}$ and $E_{3}$ edges (called $E_{4}$ ).

A directed graph ${\varsigma }$ is constructed from a 9000 bp sequence containing 10 110 nodes and $\sim $$18\,000\times (k+10)$ edges. Because of the distances between any two nodes in the graph do not exceed three, the model can easily learn the long-term dependencies of sequences.

After establishing the graph ${\varsigma }$, the representation of all nodes are updated by GSA mechanism [31] as follows: 


(1) \begin{align*} & h^{u}=\operatorname{GSA}\left(\varsigma,h^{u}\right)=\left[head_{1}^{u},\cdots,head_{h}^{u}\right]W^{O} \end{align*}

(2) \begin{align*} & {head}_{i}^{u}=\mathrm{softmax}\Bigg(\frac{{Q}_{i}^{u}{K}_{i}^{u\intercal}}{\sqrt{d}}\Bigg){V}_{i}^{u} \end{align*}

(3) \begin{align*} & Q_{i}^{u}=H_{i}W_{i}^{Q} \end{align*}

(4) \begin{align*} & K_{i}^{u}=A^{u}W_{i}^{K} \end{align*}

(5) \begin{align*} & V_{i}^{u}=A^{u}W_{i}^{V} \end{align*}

(6) \begin{align*} & A^{u}=\text{concat}\Big(\big\{h_{v}|v\in A(u)\big\}\Big). \end{align*}

$h^{u}$ is the representation of the node $u$ ; $W^{O}$ is the output weight vector; ${head}_{h}^{u}$ represents the $k$ head attention of the node $u$ in the multi-head attention. $Q_{i}^{u}$, $K_{i}^{u}$ and $V_{i}^{u}$ are query vector, key vector and value vector of the multi-head attention, respectively. is the weight of the k head attention. $W_{i}^{Q}$, $W_{i}^{K}$ and $W_{i}^{V}$ d is the dimension of vector representations of each node. $A(u)$ is the set of neighbors of a node in the graph $\varsigma $.

The relative positional coding [32] is introduced to represent the relative positional relationship of each node in the parent sequence by adding a parameter to the query vectors and key vectors [4]. 


(7) \begin{align*}& {head}_{i}^{u}=\text{softmax}\left(\frac{{Q}_{i}^{u}\left({K}_{i}^{u}+{R}^{u}\right)^{T}}{\sqrt{d}}\right){V}_{i}^{u},\end{align*}

where ${R}^{u}=\mathrm{concat}\left (\left \{{r}_{\nu ,u}|{v}\in{A}(u)\right \}\right )$ is the relative position between $A(u)$ and all nodes $v$ connected to the nodes $u$.

Each node is initialized using the trained node embedding vectors from the GCN-based node embedding network. The vectors of the 10 900-bp sequences nodes are finally merged as the order of 9000 bp sequences. The merged vectors are then fed into a fully connected network with 20 hidden units before being classified by a softmax layer. The model is trained by the training set. All parameters are updated by Adam [30] optimizer with a learning rate of 0.0002 and a batch size of 128 for 50 epochs to reduce the cross-entropy loss.

## RESULTS

### Performance of VirGrapher on the validation set

**Figure 3:**
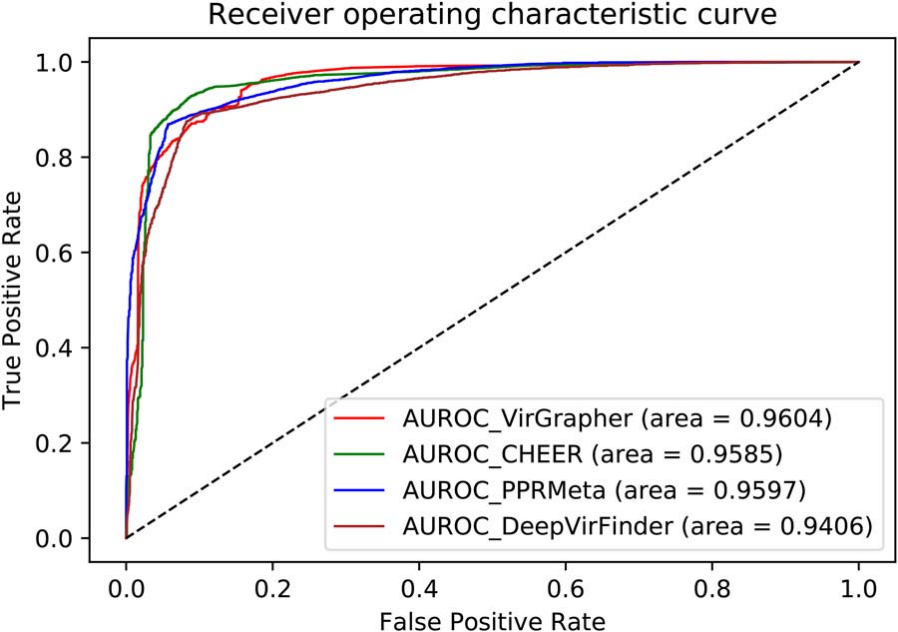
The ROC curves and AUC values of the four methods on the validation set.

VirGrapher was tested by the validation set after being fully trained. Three deep learning-based methods, PPR-Meta, CHEER and DeepVirFinder, were utilized to make a comparation (shown in Figure 3). VirGrapher obtained the highest AUC value of 0.9604, which is 0.007, 0.0019 and 0.0198 higher than PPR-Meta, CHEER and DeepVirFinder, respectively. The accuracies, recalls, precisions, specificity and F1 scores of the four methods are calcuated and made a comparison in Table 1. The values of five criteria for VirGrapher are 0.9413, 0.9437, 0.9392, 0.9389 and 0.9414, respectively, achieving the best performance among these methods.

**Table 1:** The five criteria of the four methods on the validation set. Bold values represent the best performance.

Criteria	DeepVirFinder	PPR-Meta	CHEER	VirGrapher
Accuracy	0.9263	0.9389	0.9398	**0.9413**
Recall	0.9259	0.9397	0.9410	**0.9437**
Precision	0.9265	0.9382	0.9387	**0.9392**
Specificity	0.9266	0.9381	0.9385	**0.9389**
F1 score	0.9262	0.9389	0.9389	**0.9414**

### Performance on the CAMI_high dataset

**Figure 4:**
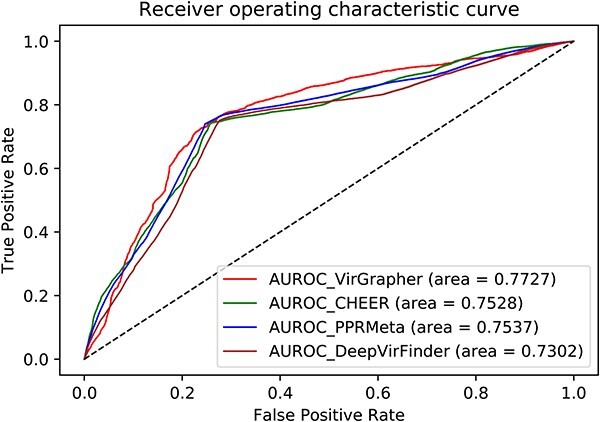
The ROC curves and AUC values of the four methods on the CAMI_high dataset.

About 732 viral sequences and 2198 host sequences from the CAMI_high dataset were input to VirGrapher and the other three benchmark methods. VirGrapher achieved the highest AUC value of 0.7727, which is 0.0190, 0.0199 and 0.0425 higher than PPR-Meta, CHEER and DeepVirFinder, respectively (shown in Figure 4). The five criteria of these methods are calculated and made a comparison in Table 2. The accuracy, recall, precision, specificity and F1 score of VirGrapher are 0.7437, 0.7418, 0.4914, 0.7443 and 0.5912, respectively, achieving a better performance than the benchmark methods.

**Table 2:** Comparison of Accuracies, Recalls, Precisions, Specificities and F1 Scores of the five methods on the CAMI_high dataset. Bold values represent the best performance.

Criteria	DeepVirFinder	PPR-Meta	CHEER	VirGrapher
Accuracy	0.7099	0.7229	0.7201	**0.7437**
Recall	0.7063	0.7281	0.7227	**0.7418**
Precision	0.4488	0.4651	0.4616	**0.4914**
Specificity	0.7111	0.7211	0.7193	**0.7443**
F1 score	0.5488	0.5676	0.5634	**0.5912**

### Experiments on the CAMI Marine dataset

**Figure 5:**
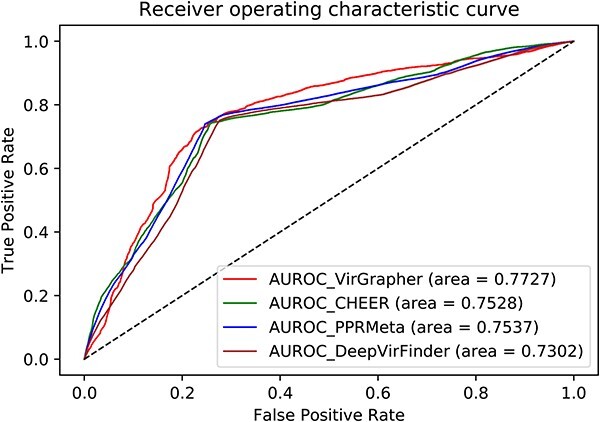
The ROC curves and AUC values of the four methods on the CAMI Marine dataset.

When tested by the CAMI Marine dataset, VirGrapher achieved an AUC value of 0.8494, which is 0.0055, 0.0192 and 0.0569 higher than PPR-Meta, CHEER and DeepVirFinder, respectively (Figure 5). The accuracies, recalls, precisions, specificity and F1 scores are calculated and made a comparison in Table 3. VirGrapher achieved the best performance, 0.8014, 0.8157, 0.7651, 0.8012, 0.7829 for the five criteria, respectively.

**Table 3:** Comparison of Accuracies, Recalls, Precisions, Specificities and F1 Scores of the five methods on the CAMI marine dataset. Bold values represent the best performance.

Criteria	DeepVirFinder	PPR-Meta	CHEER	VirGrapher
Accuracy	0.7770	0.7974	0.7938	**0.8014**
Recall	0.7832	0.7988	0.7968	**0.8157**
Precision	0.7350	0.7600	0.7552	**0.7651**
Specificity	0.7720	0.7962	0.7914	**0.8012**
F1 score	0.7583	0.7789	0.7754	**0.7829**

### Performance comparison on the real human gut metagenome

**Figure 6:**
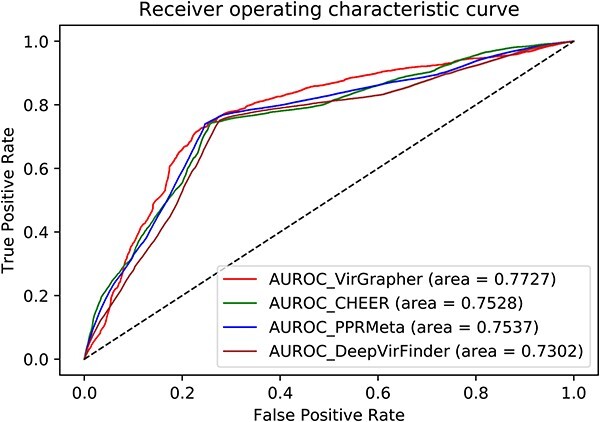
The ROC curves and AUC values of classification results on real human gut metagenome dataset.

VirGrapher was used to identify viral sequences from the real human gut metagenome. Compared with the other three methods, VirGrapher achieved the highest AUC value of 0.9193, which is 0.0159, 0.0033 and 0.0316 higher than PPR-Meta, CHEER and DeepVirFinder, respectively (shown in Figure 6). VirGrapher also achieved the best performance for the five criteria (shown in Table 4). The identification accuracy, recall, precision, specificity and F1 score of VirGrapher are 0.8590, 0.8524, 0.4783, 0.8600 and 0.6128, respectively.

**Table 4:** Comparison of Accuracies, Recalls, Precisions, Specificities and F1 Scores of the five methods on real human gut metagenome dataset. Bold values represent the best performance.

Criteria	DeepVirFinder	PPR-Meta	CHEER	VirGrapher
Accuracy	0.7995	0.8369	0.8584	**0.8590**
Recall	0.7984	0.8250	0.8482	**0.8524**
Precision	0.3751	0.4351	0.4770	**0.4783**
Specificity	0.7997	0.8387	0.8599	**0.8600**
F1 score	0.5104	0.5697	0.6106	**0.6128**

### The potential of VirGrapher to identify unknown viral species

About 1340 virus RefSeq genomes (between 13th October 2020 and 31st December 2022) were downloaded from NCBI Virus (https://www.ncbi.nlm.nih.gov/labs/virus/vssi/#/virus? SeqType_s=Genome&SourceDB_s=RefSeq). Sequences from these genomes were split into a set of non-overlapped fragments with a length of 9000 bp, resulting 4129 viral sequences. These long viral sequences were discovered after these from the training dataset and could be considered as novel ones. To verify the potential of VirGrapher to identify unknown viral species, VirGrapher and the other three benchmark methods were tested by these novel viral sequences and made a comparison in Table 5. VirGrapher identified 2016 from 4129 long viral sequences, which was the most among the four methods. To some extent, VirGrapher has the potential to identify unknown viral species.

**Table 5:** The number of correctly identified long novel viral sequences by the four methods. Bold values represent the best performance.

	DeepVirFinder	PPR-Meta	CHEER	VirGrapher
Num.	1608	1549	1754	**2016**
Accuracy	0.3894	0.3752	0.4248	**0.4883**

### The performance of VirGrapher on identifying short viral sequences

When the query sequence is as short as 500 bp, it will be zero-padded to 540 bp and be divided into six 90-mer subsequences. Then, these 90-mer subsequences are divided into several 9-mer subsequences and bases. We can still build edges of E1, E2 and E3. However, edges E4 will not exist because the query sequence is shorter than 900 bp. In this way, the new graph for 500 bp sequence is a part of the original graph in VirGrapher, where the rest parts are zeros. The nodes of subsequences are also embedded by the GCN-based node embedding network as the initialized vectors. About 1000 viral sequences and 1000 host sequences with a length of 500 bp are randomly subsampled from the validation set to build a testing dataset for identification of short viral sequences. The short query sequences from this testing dataset are input into the fully trained VirGrapher. The final vectors are also fed into a fully connected layer with 20 hidden units before being classified by a softmax layer.

The identification results are shown in Table 6. Although VirGrapher identified 1.10, 9.22 and 4.50% less viral sequences than PPR-Meta, CHEER and DeepVirFinder, respectively, the gap is acceptable considering the outperformance of VirGrapher in identifying long viral sequences. The poor performance of VirGrapher on identification of short viral sequences may be caused by the insufficient information from short sequences. The built graph for 500 bp sequences is much simpler than original graph for 9000 bp sequences.

**Table 6:** Comparison of four methods on identification viral sequences with a length of 500 bp. (Num. represents the number of correctly identified viral sequences from all 1000 viral sequences.)

Methods	PPR-Meta	CHEER	DeepVirFinder	VirGrapher
Num.	816	**889**	845	807
Accuracy	0.8025	**0.8905**	0.8480	0.7975
Recall	0.8160	**0.8890**	0.8450	0.8070
Precision	0.7945	**0.8917**	0.8501	0.7920
Specificity	0.7890	**0.8920**	0.8510	0.7880
F1 score	0.8051	**0.8903**	0.8475	0.7994

### Comparison of running time for VirGrapher and benchmark methods

We timed VirGrapher and the other three benchmark methods (PPR-Meta, CHEER and DeepVirFinder) in the strategies of testing on the validation set (10 000 viral sequences and 10 000 host sequences with a length of 9000 bp). The equipment used for analysis is Intel Core i9-13900K (CPU) with the memory of 128 Gb. The time consuming is shown in Table 7. VirGrapher has the maximum time consumption for testing on the validation set, costing 653 s. The huge time consumption of VirGrapher is caused by the strategy of GCN-based node embedding and graph-based feature extraction. DeepVirFinder and PPR-Meta translate bases into one -hot vectors before extracting features. Cheer embeds k-mer fragments by a simple word embedding model. However, VirGrapher has to train several complex GCN models layer by layer to learn meaningful node embedding matrix, which takes a lot of time. None of these methods exceed our equipment’s maximum memory (128 Gb).

**Table 7:** Comparison on time consuming of VirGrapher, CHEER, DeepVirFinder, and PPR-Meta.

Methods	CHEER	DeepVirFinder	PPR-Meta	VirGrapher
Time				
Consumption (s)	582	410	488	653

## DISCUSSION

Currently, deep learning-based methods identify long viral sequences by dividing them into a set of non-overlapped short sequences (such as 500 bp). These short subsequences are identified separately to get their own viral scores. The average of these scores is regard as the final scores which are contributed to identify if the long sequence is viral. However, these short subsequences contribute independently to the final decision, omitting the natural relationships from the long sequence. This may lead to poor performance of deep learning methods in identifying long viral sequences. VirGrapher works better than current benchmark deep learning methods by constructing relationships among short subsequences from long ones. Firstly, the GCN-based node embedding network learns the relationships between each part of a long sequence by training a GCN model for every level. Secondly, a graph containing multilayer connections is constructed from a long sequence. These connections make the distances between any two nodes in the graph do not exceed three, and make the model easily learn the long-term dependencies of sequences and strengthens effective links between short subsequences. Thirdly, the graph-based classifier further learns the positional relationships and features of short subsequences from long ones by GSA mechanism and relative positional coding.

To verify the effectiveness of layer-by-layer embedding from the GCN-based node embedding network and effective feature learning from the graph-based classifier in VirGrapher, a set of comparative experiments are conducted. Firstly, instead of layer-by-layer embedding, only GCN_4 (containing the nodes of 9-mer parent sequences and nodes of bases initialized by one-hot vectors) is trained by ETD_4 to generate initial node embeddings. The embeddings of the long sequence nodes are generated by concatenation of related 9-mer nodes. Then, the embedded graph is trained by the graph-based classifier with the same parameters (namely Variant_1). Secondly, a common two-layer GCN model is used to learn graph features instead of the graph-based classifier in VirGrapher (namely Variant_2). These two variant models (Variant_1 and Variant_2) are trained by the training set and tested by the validation set as VirGrapher. The identification results are shown in Table 8. When the way of embedding is changed, the identification performance declines rapidly, meaning that layer-by-layer embedding could learn the relationships between each layer of subsequences. The selection of GCN model or GSA model impacts less on learning graph features.

**Table 8:** The identification results of VirGrapher, Variant_1 and Variant_2 on the validation set. Bold values represent the best performance.

Criteria	VirGrapher	Variant_1	Variant_2
Accuracy	**0.9263**	0.7679	0.8704
Recall	**0.9259**	0.7825	0.8679
Precision	**0.9265**	0.7603	0.8723
Specificity	**0.9266**	0.7533	0.8729
F1 score	**0.9262**	0.7712	0.8701

VirGrapher compensates for the loss of correlations between subsequences when a long viral sequence is identified by existing deep learning-based methods, improving the accuracy of identifying long viral sequences. It supplements viral analysis since most existing methods tend to deal with long sequences generated from assembling and binning. Moreover, identifying viral sequences is the very first step in the flow of viral analysis. The improvement of the identification accuracy could have an impact on downstream work, such as finding associations between temperate phages or their signatures and CRC. We hope that VirGrapher could play an important role in the realm of virus analysis.

**Key Points**

- Correctly identifying viruses from these mixed sequences is critical in viral analyses. When identifying a long sequence, existing deep learning-based methods divide the long sequence into several short subsequences and identify them separately. This makes the relationships between them be omitted, leading to poor identification performance. VirGrapher is proposed to solve the problem.
- VirGrapher contains a GCN-based node embedding network and a graph-based classifier. The GCN-based node embedding network learns the relationships between each part of a long sequence by training a GCN model for every level. A graph containing multilayer connections is constructed from a long sequence. These connections make the distances between any two nodes in the graph do not exceed three, and make the model easily learn the long-term dependencies of sequences and strengthens effective links between short subsequences.
- Trained by 13 274 virus RefSeq genomes, VirGrapher outperforms three benchmark methods (DeepVieFinder, PPR-Meta, and CHEER) for identifying long viral sequences. The improvement could have an impact on downstream work, such as viral taxonomy, viral host prediction, and so on.

## Data Availability

The virus RefSeq genomes can be found from NCBI Virus (https://www.ncbi.nlm.nih.gov/labs/virus/vssi/#/virus?SeqTypes=Genome&SourceDBs=RefSeq). The host RefSeq genomes from VirFinder (DOI 10.1186/s40168-017-0283-5). The CAMI high dataset can be found at https://data.cami-challenge.org/camiClient.jar. The CAMI Marine dataset can be found at https://data.cami-challenge.org/participate. The real human gut metagenome dataset can be found from NCBI [SRA052203].

## Author Biographies

**Yan Miao** is an associate professor of College of Computer and Control Engineering, Northeast Forestry University. His research focuses on bioinformatics in viral metagenomes and related diseases using deep learning methods.

**Zhenyuan Sun** is a master’s student of College of Computer and Control Engineering, Northeast Forestry University. His research focuses on computational methods and tools development for metagenomes.

**Chenjing Ma** is a master’s student of College of Computer and Control Engineering, Northeast Forestry University. Her research interest is gene detection and metagenomic data analysis.

**Chen Lin** is a professor of National Institute for Data Science in Health and Medicine, Xiamen University. She focuses on artificial management of large-scale unstructured or semi-structured data and bioinformatics.

**Guohua Wang** is a professor at the College of Information and Computer Engineering, Northeast Forestry University. He is also a Principal Investigator at Key Laboratory of Tree Genetics and Breeding, Northeast Forestry University. His research interests are bioinformatics, machine learning and algorithms.

**Chunxue Yang** is an associate professor of College of Landscape Architecture, Northeast Forestry University. She focuses on soil microbiome and biological data analysis.
